# Low-Power Sonication Can Alter Extracellular Vesicle Size and Properties

**DOI:** 10.3390/cells10092413

**Published:** 2021-09-14

**Authors:** Zubair Ahmed Nizamudeen, Rachael Xerri, Christopher Parmenter, Kiran Suain, Robert Markus, Lisa Chakrabarti, Virginie Sottile

**Affiliations:** 1 School of Medicine, The University of Nottingham, Nottingham NG7 2RD, UK; mszzn@exmail.nottingham.ac.uk; 2 School of Pharmacy, The University of Nottingham, Nottingham NG7 2RD, UK; rachael.xerri@nottingham.ac.uk (R.X.); christopher.parmenter@nottingham.ac.uk (C.P.); kiransuain@outlook.com (K.S.); 3 School of Life Sciences, The University of Nottingham, Nottingham NG7 2RD, UK; robert.markus@nottingham.ac.uk; 4 School of Veterinary Medicine and Science, Sutton Bonington Campus, The University of Nottingham, Loughborough LE12 5RD, UK; lisa.chakrabarti@nottingham.ac.uk; 5 Department of Molecular Medicine, The University of Pavia, 27100 Pavia, Italy

**Keywords:** extracellular vesicles, stem cells, sonication, cellular uptake

## Abstract

Low-power sonication is widely used to disaggregate extracellular vesicles (EVs) after isolation, however, the effects of sonication on EV samples beyond dispersion are unclear. The present study analysed the characteristics of EVs collected from mesenchymal stem cells (MSCs) after sonication, using a combination of transmission electron microscopy, direct stochastic optical reconstruction microscopy, and flow cytometry techniques. Results showed that beyond the intended disaggregation effect, sonication using the lowest power setting available was enough to alter the size distribution, membrane integrity, and uptake of EVs in cultured cells. These results point to the need for a more systematic analysis of sonication procedures to improve reproducibility in EV-based cellular experiments.

## 1. Introduction

Extracellular vesicles (EVs) are lipid-membraned nanovesicles secreted by biological cells [1]. EVs store and transport a variety of proteins and nucleotides in a cell-specific manner and are therefore considered to be important modulators of cell-cell communication in vivo [1,2]. EVs have raised increasing biomedical interest as potential biomarkers for diverse pathological states such as cancer, through the analysis of their protein and RNA cargo [3,4,5,6,7]. In a recent study, an EV-based antibody array profiling identified CD151, CD171, and Tetraspanin 8 among the most significant molecules to differentiate histological lung cancer patients from cancer-free individuals [8]. EVs are also considered a potential therapeutic resource as a drug cell delivery vector based on their efficient cellular uptake [6,7,9,10], as recently illustrated by intravenous administration of engineered EVs loaded with curcumin able to target an ischemic brain lesion in rodents [11].

In vitro isolated EVs are prone to aggregating, a factor suggested to result in technical challenges for subsequent particle characterization [12,13], leading to variability in measurements of particle size and concentration. Low-power sonication has been widely used to promote the disaggregation of isolated EVs prior to analysis [14,15]. The technique separates aggregates by vibrating particles in a suspension [14,15]. Aside from the dissemination of aggregates, sonication can be used for a variety of purposes, including degradation and disruption of cellular structures such as biological membranes, chromatin, and DNA [16,17]. The effect of sonication on membranes has also been exploited to facilitate lysis [18] and transfection [19] of isolated EVs. However, the effect of this sonication step on particle size and EV structural integrity remains unclear. Given the notable physical effects of sonication on particles, it is necessary to investigate the effect of low-power sonication on isolated EVs beyond disaggregation. Additionally, the use of ‘low-power’ modes on different sonication instruments can produce varying final power outputs, which reduces the reproducibility of observations in EV research. In this study, the effect of low-power sonication on stem cell-derived EVs was investigated by analysing the size distribution, structure, concentration, and cellular uptake of sonicated EVs in vitro.

## 2. Materials and Methods

All materials were purchased from Thermo Fisher Scientific (Loughborough, UK) unless otherwise stated.

### 2.1. Cell Culture and Staining

Mouse mesenchymal stem cells (MSC) were cultured as previously described [20,21,22] in a culture medium containing low-glucose Dulbecco’s modified Eagle’s medium supplemented with 10% fetal bovine serum (FBS), 1% penicillin/streptomycin, 1% L-glutamine and 1% non-essential amino acids. Primary mouse neural stem cells (NSCs) were seeded onto Geltrex coated 96-well plates and differentiated as previously described [23]. Cell phenotype was confirmed by immunohistochemistry as previously described [20,23] using primary antibodies for mouse βIII-Tubulin (1:1000 dilution, Ab18207, Abcam, Cambridge, UK) and mouse GFAP (1:250 dilution, MA515086) and secondary antibodies Alexa Fluor 488 anti-rabbit (1:500 dilution, A11008) and Alexa Fluor 594 (1:500 dilution, A11001), using a Zeiss Elyra PS.1 microscope (ZEISS, Cambridge, UK) equipped with C-Apochromat 63×/1.2 W Korr M27 objective for confocal imaging followed by ImageJ thresholding.

### 2.2. EV Labelling and Isolation

Labelling and one-step isolation of EVs were performed as previously described [20]. Cells were cultured in a T175 flask (for MSCs) or T75 flasks (for NSCs) for EV isolation. Briefly, cells were washed with Phosphate Buffered Saline (PBS) and incubated with EV enrichment medium (MSC culture medium containing Exo-free FBS (System Biosciences) instead of FBS, with added DiD Vybrant Cell labelling solution (5 µL/mL) according to manufacturer’s instructions. DiD binds to lipid structures allowing EVs to be labelled prior to isolation. After 6 h, the medium was filtered using 0.45 µm syringe filters (SLS, Nottingham, UK) and processed with the exoEasy Maxi Kit (Qiagen, Manchester, UK) according to the manufacturer’s instructions. Briefly, samples were diluted in XBP buffer and spun down using exoEASY Maxi columns. Column bound EVs were washed using XWP wash buffer, centrifuged to remove excess dye, and eluted in XE buffer.

### 2.3. Low-Power Sonication of EVs

Freshly isolated EVs were split into two groups—‘non-sonicated’ (nsEVs) and ‘sonicated’ (sEVs). nsEVs were subjected to gentle manual tapping and pipetting up and down 3 times prior to analysis (using a 1000 µL pipette tip). sEVs were subjected to low-power sonication using Bioruptor (Diagenode, Seraing, Belgium) 230 V/2.1 A (EU) 50/60 Hz ‘Low-power’ option, 10-s on-off cycle for 3 times. For MSC-derived EVs, samples were added to Pall Omega 300 K nanosep membrane (Pall, Portsmouth, UK) and centrifuged at 5000× *g* for 10 min to concentrate to 90 µL final. EVs were stored at −80 °C and used within 7 days.

### 2.4. Dynamic Light Scattering (DLS)

A Zetasizer Nano ZS (Malvern Panalytical, Malvern, UK) was used to determine particle hydrodynamic size. Measurements were taken at 173° backscatter with auto attenuation (3 measurements/sample).

### 2.5. Nanoparticle Tracking Analysis (NTA)

Sample preparation was performed as previously described [20]. An LM10/14 Nanosight (Nanosight, Malvern Panalytical, Malvern, UK) instrument was used to analyse EVs. Prior to analysis, a 1:10 dilution of CPC100 (IZON) and a 1:1000 dilution of 200 nm polystyrene (Malvern Panalytical, Malvern, UK) nanoparticles were used to test the sensitivity of the instrument. EV samples were diluted such that less than 200 particles were tracked per image. Automatic settings were applied for the minimum expected particle size, minimum track length, and blur settings. For capture settings, screen gain was set at 1, and camera level was set at 15 (shutter 1500; gain 574). For analysis settings, screen gain was set at 10, and the detection threshold was set at 3. Five 60-s movies were captured at 30 frames per second for each sample. Data processing and analysis of particle size distribution were performed using NTA Software 3.3 Dev build 3.3.301 (https://www.malvern.com) (accessed on 8 October 2018).

### 2.6. Stochastic Optical Reconstruction Microscopy (d-STORM)

d-STORM was performed as previously described [20] using a Zeiss Elyra PS.1 super-resolution microscope, with α-Plan Apo 100×/1.46 oil immersion objective in total internal reflection microscopy (TIRF) mode. Before the STORM experiment, a wide-field snapshot was captured using the ‘16 Avg’ option at 0.5% (0.02 kW/cm^2^) laser power in TIRF mode. Then d-STORM was run for 10,000 frames at 8% (0.31 kW/cm^2^) laser power, with 35 ms exposure, and 200 gain on the EM-CCD iXon Du 897 camera (Andor, Belfast, UK). Images were processed using the PALM module of the Zeiss Zen Black software. The number of photons threshold was adjusted to 200 and a value of 0.25 PSF was used. Image J macro (https://imagej.nih.gov/ij/) (accessed on 11 October 2018) was used to determine the size and concentration of EVs, as previously described [20].

### 2.7. Transmission Electron Microscopy (TEM)

Sample preparation was performed as previously described [20]. Samples were fixed with 4% paraformaldehyde (PFA), added (5 µL/grid) to glow discharged (10 s at 5 mA using an Agar turbo coater aux power unit and dedicated glow discharge head) carbon (lacey/holey) EM grids (EM resolutions, Sheffield, UK) and adsorbed for 20 min. Samples grids were then washed with PBS, incubated with 1% glutaraldehyde for 5 min, washed with sterile distilled water, and incubated with 3% uranyl-acetate for 15 min for negative staining. TEM was carried out using a Tecnai Biotwin-12 with an accelerating voltage of 100 kV.

### 2.8. Cryo-TEM

Sample preparation for cryo-TEM was adapted from an existing protocol [20]. Glow discharged (10 s at 5 mA using an Agar turbo coater aux power unit and dedicated glow discharge head) Holey carbon copper TEM grids were used (EM resolutions, Sheffield, UK). Samples were left to adsorb onto the grids (5 µL/grid) for 2 min, excess solution was removed using a filter, and samples were frozen using a Gatan CP3 plunge freezing unit (Ametek, Leicester, UK), blotted for 1 s and frozen in liquid ethane. Samples were loaded into a Gatan 626 cryo-TEM holder on a JEOL 2100+ TEM (Jeol, Welwyn Garden City, UK) and imaged acquired for 2–4 s at a dose of below 10 e/A^2^, using a US1000 CCD camera and Digital Micrograph GMS 3 software.

### 2.9. Flow Cytometry

DiD-labelled EVs (sonicated or non-sonicated) were added to MSCs at an equal volume (0.5 µL of EVs/cm^2^) or at an equal particle count (1 × 10^9^ particles/cm^2^) for a 6 h incubation followed by a gentle PBS wash, cell trypsinisation and centrifugation at 200× *g* for 5 min. Cells resuspended in PBS were analysed on a Cytomics FC 500 (Beckman Coulter, Brea, CA, USA) with a minimum of 50,000 events recorded/sample, using FlowJo V10.5 software (https://www.flowjo.com/, accessed on 8 November 2018).

### 2.10. Statistical Analysis

All experiments were conducted in triplicates. Data is presented as mean ± SD with statistical significance level set for * *p* < 0.05, ** *p* < 0.01, *** *p* < 0.001, **** *p* < 0.0001. Histograms and scatter plots were made using Prism (GraphPad software version 8, San Diego, CA, USA, http://www.graphpad.com) (accessed on 12 October 2018) unless otherwise stated. Unpaired two-tailed student’s t-test was run to determine the statistical significance for flow cytometry, d-STORM, and immunodetection experiments, with Shapiro-Wilk test and D’Agostino & Pearson test (depending on sample number) used to test for normal distribution.

## 3. Results and Discussion

### 3.1. TEM Analysis of Sonicated and Non-Sonicated EVs

**Figure 1:**
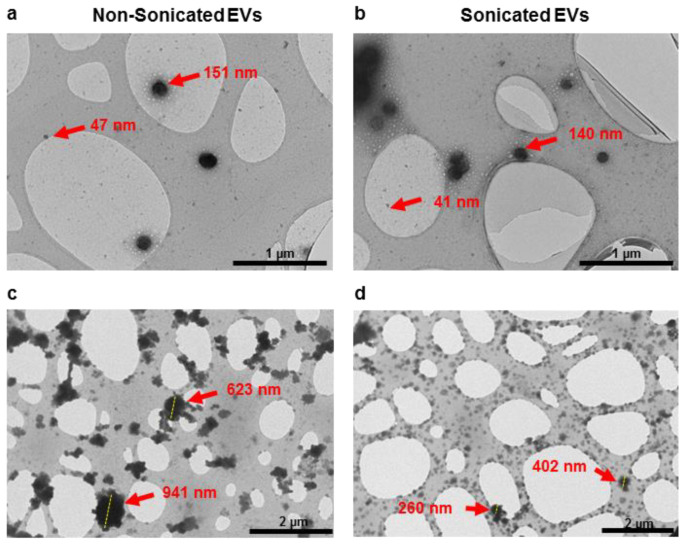
TEM imaging showing particle distribution of non-sonicated (**a**,**c**) and sonicated (**b**,**d**) EV isolates observed at higher magnification (≥26.5 k, (**a**,**b**)) and lower magnification (≤11.5 k, (**c**,**d**)).

To investigate the effect of sonication on EV disaggregation, samples were analysed under transmission electron microscopy (TEM). Observation at higher magnification (≥26.5 k) showed both samples contained EVs of sizes of 30 to 200 nm (Figure 1a,b). At a lower magnification (≤11.5 k), TEM images showed that sonication resulted in a lower frequency of large aggregates on EM grids, as compared to non-sonicated samples (Figure 1c,d). These results suggested that low-power sonication promoted disaggregation of particles visualised under conventional TEM.

### 3.2. Particle Size Distribution Analysis of Sonicated and Non-Sonicated EVs by DLS, NTA and d-STORM

**Figure 2:**
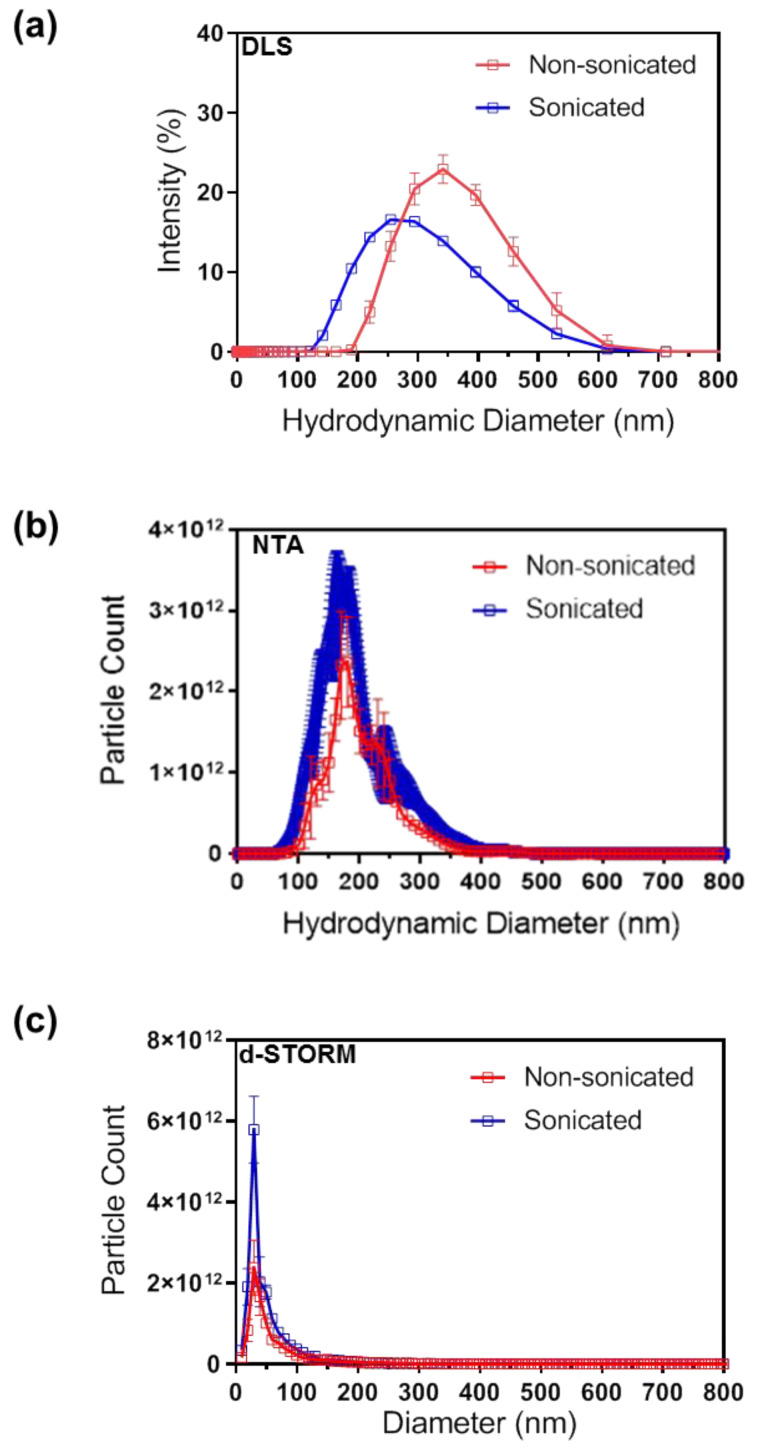
Particle size distribution analysis of non-sonicated and sonicated EVs by (**a**) DLS, (**b**) NTA and (**c**) d-STORM. N = 3, data presented as mean ± SD.

Changes in EV particle size distribution after sonication were analysed with DLS and NTA techniques, which detect the Brownian motion of particles in a suspension and derive their hydrodynamic diameter using the Stokes-Einstein equation [24,25]. DLS highlighted an increase in the proportion of particles below 200 nm in sEVs compared to nsEVs (Figure 2a). Similarly, NTA also detected an increase in the proportion of particles below 200 nm in sEVs compared to nsEVs (Figure 2b). The recently described EV characterisation technique based on d-STORM imaging, which enables the size and concentration measurements of fluorescently labelled EVs, was also used [20]. d-STORM analysis (Figure 2c) detected a significant increase in particles below 50 nm in sEVs ((1.18 ± 0.057) × 10^13^ particles/mL) compared to nsEVs ((0.61 ± 0.055) × 10^13^ particles/mL) (*** *p* = 0.0002), and a significant increase in total EV particle count in sEVs ((1.68 ± 0.066) × 10^13^ particles/mL) compared to nsEVs ((0.94 ± 0.074) × 10^13^ particles/mL) (*** *p* = 0.0002).

DLS, NTA, and d-STORM detected a reduction in the average EV size post-sonication, although the absolute size values varied between techniques (Table 1) in line with previous studies [20]. This variability could be expected as light scattering techniques including NTA tend to overestimate the proportion of larger size EVs, possibly due to the fact that these scatter more light, which results in the under-representation of smaller EVs in a highly polydispersed sample [24]. In contrast, d-STORM allows direct visualisation of single particles and subsequent size and count analysis of polydispersed samples with high resolution, sensitivity, and reproducibility [20]. Here, the d-STORM analysis revealed a significant decrease in the average particles size in post-sonication samples (Table 1). Differences in variation range between d-STORM and other standard techniques such as NTA, qNano, flow cytometry, and TEM, should also be considered when selecting the approach to establish EV size and concentration [26].

Taken together, these results confirmed that low-power sonication increased the number of smaller particles in EV samples, particularly in the population below 50 nm in size. It is however unclear whether sonication results in the breakdown of larger particles into multiple smaller particles, or whether aggregates are disassembling into lone particles in suspension; both possibilities are compatible with the observed particle size distribution and concentration data.

### 3.3. Structural Analysis of Sonicated and Non-Sonicated EVs by Cryo-TEM

**Figure 3:**
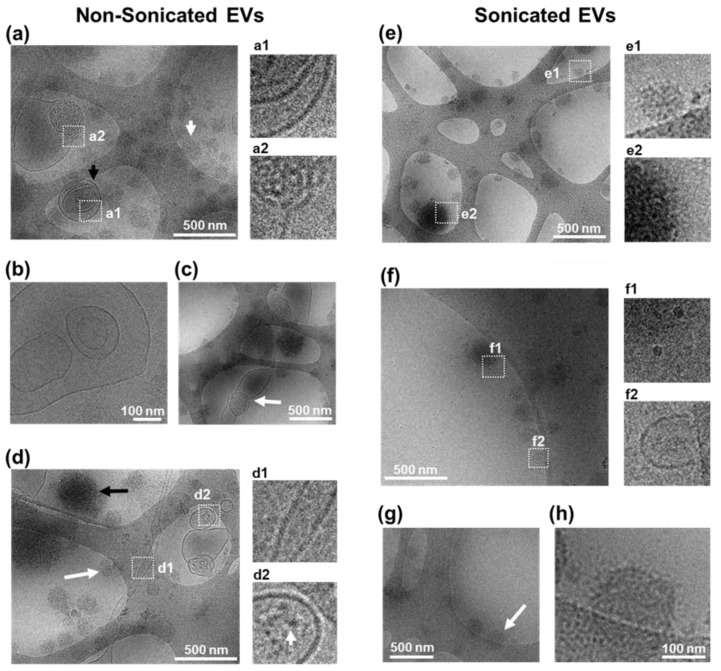
Cryo-TEM characterisation of EVs. (**a**–**d**) Characterisation of non-sonicated EVs. (**a**) Example of multiple EVs with (black arrow) or without (white arrow) a clear membrane border. Inserts show magnified area of EV containing multiple concentric EVs (**a1**), and of EV with membrane disorganisation (**a2**). (**b**) View of a multi-vesicular non-concentric EV. (**c**) Image from an EV structure >500 nm with disorganised shape and membrane (white arrow). (**d**) Image showing a tubular EV-structure with defined border (**d1**) and an EV with electron-dense features inside (**d2**). Arrow in (**d2**) magnified view shows features with electron contrast within an EV. Arrows in (**d**) show round structures with no clear membrane either with high electron contrast (black arrow) or low electron contrast (white arrow). (**e**–**h**) Characterisation of sonicated EVs. (**e**) Image showing multiple EVs without a clear membrane border having either low electron contrast (**e1**) or high electron contrast (**e2**). (**f**) Image showing multiple 20 nm sized EVs with border-like features (**f1**) and a portion of an EV structure with clear membrane border (**f2**). (**g**,**h**) Example of spherical structures with distorted appearance of membrane border (arrow). (**h**) Higher magnification image of selected object in ((**g**), arrow) showing punctate low contrast membrane border.

The TEM performed earlier in this study illustrated the effect of disaggregation. Under high magnification, particles were found in the typical size range for EVs, however, the cup-shaped morphology was not always clearly visible (Figure 1a,b). A recent study has reported that the cup-shaped appearance of EVs under TEM can be diversely visible due to variability in the process of fixation and negative staining, and showed that differences in sample source and/or the presence of contaminants can also play a role [27]. Here cryo-TEM was performed to visualise the lipid bilayer ultrastructure of EVs [28,29,30] and further confirm the presence of EVs in the samples analysed (Figure 3). The immunodetection of EV enrichment proteins such as CD63 should be used in the future to add further validation to complement EV structure [31].

To investigate the structure of EV post sonication, cryo-TEM was performed for morphological characterisation and membrane visualisation in their near-native state since cryo-TEM avoids fixing and dehydration of samples [28,29]. Cryo-TEM imaging showed nsEV samples as a heterogeneous population with respect to size, shape, contrast, and the presence of a defined border (Figure 3a–d). nsEVs were observed to contain spherical structures with visible lipid bi-layer membrane (referred from here as vesicles) and without visible membranes resembling lipoproteins (referred to as lipoprotein-like structures) (Figure 3a), as observed in published reports [28]. Some of these lipoprotein-like particles were observed to measure up to 400 nm (Figure 3c,d), which is larger than what has been previously observed during EV isolation from plasma samples [28]. Due to high-electron density, it is unclear if any vesicular membrane was present, pointing to the need for further assessment by lipoprotein isolation and proteomic quantification to refine their nature. nsEV samples contained vesicles of 100 to 500 nm in size, which were singular, multi-vesicular concentric (Figure 3a), or multi-vesicular non-concentric (Figure 3b) in nature with clear visibility of each membrane layer. Vesicles bigger than 500 nm with non-spherical shapes were also observed (Figure 3c), suggesting that EVs may be semi-fluid in nature, as is common with other lipid structures, which would enable particles to pass through a 0.45 µm pore whilst maintaining overall surface area and volume. Additionally, tubular structures were observed with membrane features over 2 µm in length (Figure 3d). These results suggested that accurate EV diameter estimation by DLS and NTA techniques may be limited to spherical EVs. Some vesicles contained spot-like features suggesting the presence of electron-dense cargo (Figure 3d). The lipoprotein-like structures observed in nsEVs showed differences in contrast and size, indicating heterogeneity within non-lipid bi-layered structures (Figure 3d).

Similar to nsEVs, sEVs contained a heterogeneous population of lipoprotein-like structures (Figure 3e), and smaller vesicles of 20 nm in size were observed (Figure 3f). However, in contrast to nsEVs, larger vesicles were rarely found (Figure 3e,f), suggesting that sonication resulted in a reduced vesicle size. In some 200 nm vesicular structures, a membrane border with low contrast and a punctate appearance was noted, pointing to possible membrane damage (Figure 3g,h). Line profiles across EV membrane confirmed irregular spacing, suggesting loss of membrane integrity in sEVs compared to nsEVs (Supplementary Figure S1). These results suggested that low-power sonication could have damaging effects on lipid bi-layered EVs, resulting in a bias in the sonicated EVs analysed towards lipoprotein-like structures [28,29,32]. Subsequent studies involving immunogold labelling for cryo-TEM could provide additional information on vesicle and lipoprotein heterogeneity, while density cushion ultracentrifugation and size exclusion chromatography may further improve the purity of EV isolation from biological samples [32].

Compared to TEM assessment, EV aggregation was less evident under cryo-TEM, possibly due to differences in sample preparation. For TEM, the fixation and embedding of EVs onto TEM grids, with manual blotting improved sample quantity for assessment, as compared to cryo-TEM, where the automated blotting and plunge freezing could have resulted in a loss of sample, reducing visible aggregates. TEM and cryo-TEM images were not used for EV quantification purposes in this study due to possible technical biases, for instance, the use of blotting paper to remove excess liquid from TEM grids during sample preparation could cause sample loss, protein denaturation, protein aggregation, and grid damage [33]. Furthermore, EVs can be unevenly distributed on a TEM grid, which could increase variability in EV analysis [27]. By contrast, d-STORM and NTA sample processing procedures involve unfixed EVs spread evenly across a dish or flow chamber, respectively, more representative sample distribution for quantification purposes [20,24].

Recent studies have reported that in addition to EV yield, the EV isolation procedure selected can impact the presence of lipoprotein and protein aggregate contaminants [34], and it is unclear whether changes in the concentration of such contaminants may influence the sonication-induced alteration of EVs. Supplemental quantitative analysis of associated protein and lipid components alongside particle number will be useful to refine the evaluation of sample purity. In this study, EVs were isolated using the Qiagen exoEasy Maxi kit, a widely used alternative to ultracentrifugation to streamline EV isolation from biological fluids [35,36,37]. To preserve intact EVs for multiple analyses including size distribution, ultrastructural and cell uptake analysis, the EVs were stored at −80 °C and used within seven days of storage, as previously used in multiple studies [38,39,40], and were only thawed once prior to experimental use to avoid storage induced alterations. This protocol used in the literature [35,36,37,38,39,40] yielded intact EVs as confirmed by cryo-TEM to analyse the effect of low-power sonication. Future research will now move to systematically assess whether sonication induces similar alterations in different EV samples freshly isolated through any of the common techniques including ultracentrifugation and size exclusion chromatography.

### 3.4. Cellular Uptake Analysis of Sonicated and Non-Sonicated EVs

**Figure 4:**
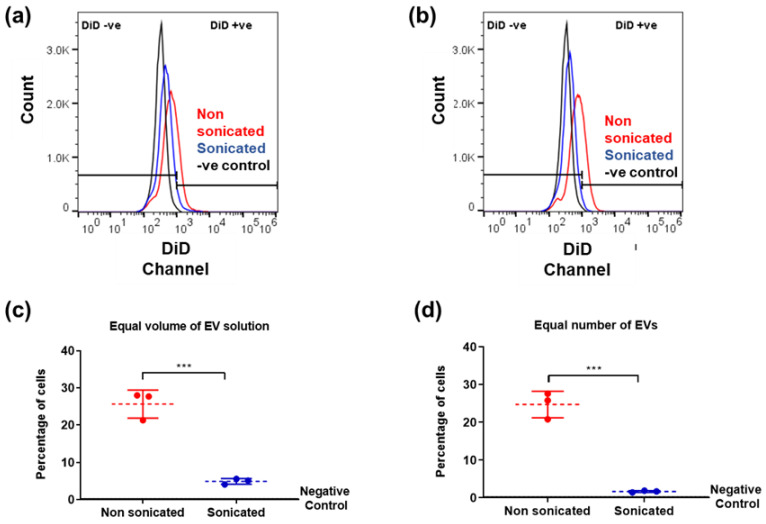
Flow cytometry analysis of EV uptake. DiD labelled EVs added at 0.5 µL/cm^2^ (**a**,**c**) or 1 × 10^9^ particles/cm^2^ (**b**,**d**). (**a**,**b**) Fluorescence detected in live cells after EV incubation compared to negative control (MSCs with no EVs added). (**c**,**d**) Quantification of cells containing EVs after incubation (red: non sonicated, blue: sonicated samples). Data analysis was performed with FlowJo v10.5 software (https://www.flowjo.com/) (accessed on 8 November 2018). N = 3, unpaired students t-test, two-tailed non-parametric; *** *p* < 0.001.

Cryo-TEM observations have confirmed structural changes associated with EVs after the low-power sonication step. To investigate whether these changes in EV size and morphology are linked to functional changes, MSCs were incubated for 6 h with nsEVs or sEVs, using a constant volume (0.5 µL/cm^2^ as 0.5× of EV isolate) of EVs per well. MSCs incubated with sEVs showed lower fluorescence intensity and lower population of DiD-positive cells compared to MSCs incubated with nsEVs (*** *p* = 0.007) (Figure 4a,c). Since d-STORM analysis indicated that sEVs contained more particles per volume than nsEVs, the experiment was repeated using the same number of particles (1 × 10^9^ particles/cm^2^) per well. Here again, MSCs treated with sEVs showed a lower fluorescence intensity and a lower population of DiD-positive cells than those exposed to nsEVs (*** *p* = 0.003) (Figure 4b,d). These results suggested an alteration of the sample properties illustrated by the reduced uptake of DiD particles with sample sonication.

**Figure 5:**
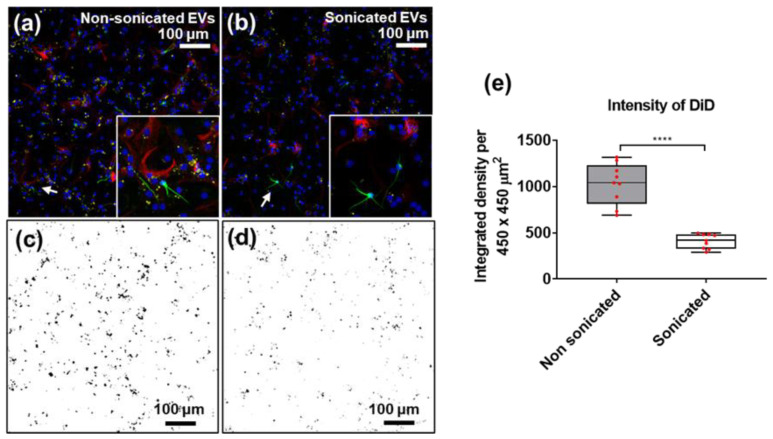
Immunofluorescence images of NSC-derived EV (DiD, yellow) uptake by differentiating neural cells labelled with βIII-Tubulin (green) and GFAP (red) at day 10, with DAPI (blue) used as nuclear counterstain. Differentiating neural stem cells incubated with (**a**) non-sonicated and (**b**) sonicated NSC-derived EVs (white arrows point to areas shown in inserts). ImageJ thresholding of DiD channel for (**c**) non-sonicated (in (**a**,**d**)) sonicated (in (**b**)) EVs taken up by cells in culture. (**e**) Quantitation of DiD intensity in (**c**,**d**) (red dots represent individual fields of view); N = 9, unpaired students t-test, two-tailed non-parametric; **** *p* < 0.0001. Image J (https://imagej.nih.gov/) (accessed on 16 December 2018) was used for thresholding (Min 40, Max 255).

To assess whether sonication also affected the uptake of EVs from different cellular origins, DiD-labelled EVs isolated from cultured NSCs were added to cultures of differentiating NSCs (Figure 5). NSC-derived EVs were added at a constant concentration (1.3 µL/cm^2^ as 0.1× of EV isolate) on days four, six, and eight of differentiation. Samples were fixed on day 10 (endpoint) and the resulting uptake was measured using confocal imaging, which showed that incubation with sEVs resulted in significantly lower DiD intensity, compared to the nsEV counterpart, indicating reduced cellular uptake of sonicated EVs (**** *p* < 0.0001). Cells with no added EVs were used as negative controls and confirmed the absence of detectable autofluorescence in the DiD channel (Supplementary Figure S2).

Taken together, the above results indicate that sonication of EVs derived from MSC and NSCs can alter their cellular uptake efficiency in recipient cells. However, the nature of the resulting functional changes associated with sonication of EVs remains undefined and calls for future research. In particular, the specific changes in cargo composition and cellular uptake of EVs post-sonication may vary between different sources of EVs and between different recipient cells, and therefore, such a study should comprehensively assess a range of samples according to published guidelines [10,41]. Investigation of whether low-power sonication of EVs induces changes in the mechanism of EV uptake by recipient cells, and the heterogeneity of isolated EVs, should be made. This can be done by immunodetection for specific markers such as EV membrane proteins (such as CD63, CD9, and CD81) and endosomal machinery markers (ESCRT, Syntenin, and ALIX) followed by TEM/cryo-TEM [31,42], correlative light electron microscopy [43,44] and/or super-resolution fluorescence microscopy [20,45].

While the present study showed that sonication using the lowest power setting altered EV properties, the systematic investigation of varying sonication power on EV integrity remains to be carried out to determine whether a threshold of sonication power, pulse, and/or frequency can be identified to achieve EV disaggregation without significant alteration of their biophysical and cell uptake properties. The findings from this study call for additional investigation exploring the use of chemical techniques to prevent aggregation such as a trehalose treatment [12], to provide additional controls and investigate alternatives to sonication for EV processing.

## 4. Conclusions

This study highlights the need to carefully consider the optimisation and standardisation of sonication parameters routinely used to disseminate aggregates for extracellular vesicle analysis. These results show that in addition to disaggregation, low-power sonication can induce significant changes in the size distribution, membrane integrity, and cellular uptake of EVs. Sonication could thus introduce variability in the characterisation of EV populations and could thus compromise the outcome of subsequent preclinical and clinical applications. The present observations warrant future work analysing the functional effects of sonication protocols on EV cargo, uptake dynamics, and receptor-based response. Additional research is thus needed to reach a consensus on defined sonication conditions that promote disaggregation without altering the biophysical properties of EVs.

## Figures and Tables

**Table 1:** Average EV size determined by DLS, NTA and d-STORM measurements; N = 3; unpaired two-tailed parametric student’s t-test.

Measurement (nm)	Non-Sonicated EVs	Sonicated EVs
DLS (mean hydrodynamic diameter ± SD)	346.9 ± 1.5	287.1 ± 4.1 (**** *p* < 0.0001)
NTA (mean hydrodynamic diameter ± SD)	202.7 ± 1.95	196 ± 4.81 (*p* = 0.0881)
d-STORM (mean diameter ± SD)	58.8 ± 2.8	48.1 ± 1.6 (** *p* < 0.01)

## Data Availability

Available upon reasonable request.

## Supplementary Materials

The following are available online at https://www.mdpi.com/article/10.3390/cells10092413/s1, Figure S1: EV membrane integrity analysis post sonication. Image J line profile snapshots across EV membrane from cryo-TEM images and corresponding line profiles of (a,b) non-sonicated and (c,d) sonicated samples, Figure S2: Immunofluorescence images of no EV control showing differentiating neural cells in vitro.
