# Supplementary material for: Low-Power Sonication Can Alter Extracellular Vesicle Size and Properties
Source: Cells. 2021 Sep 14;10(9):2413. doi: 10.3390/cells10092413 (PMC8466153; doi:10.3390/cells10092413)
Supplement: Supplementary file 1 [file cells-10-02413-s001.zip › cells-1332364-supplementary.pdf]

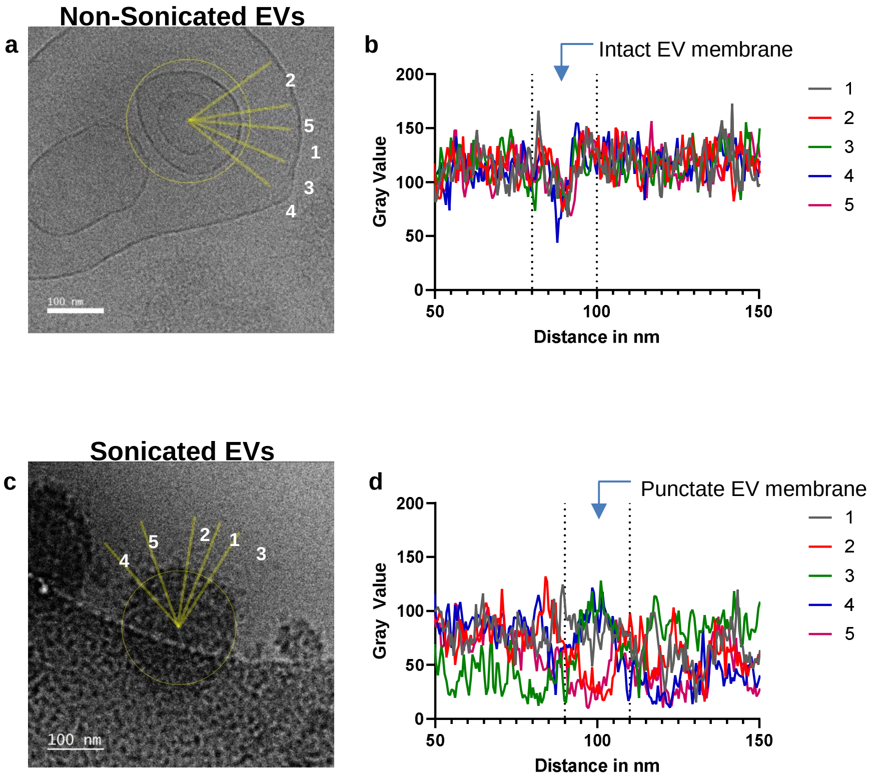


**Supplementary Figure S1**: EV membrane integrity analysis post sonication. Image J line profile snapshots across EV membrane from cryo-TEM images and corresponding line profiles of (a, b) non-sonicated and (c, d) sonicated samples

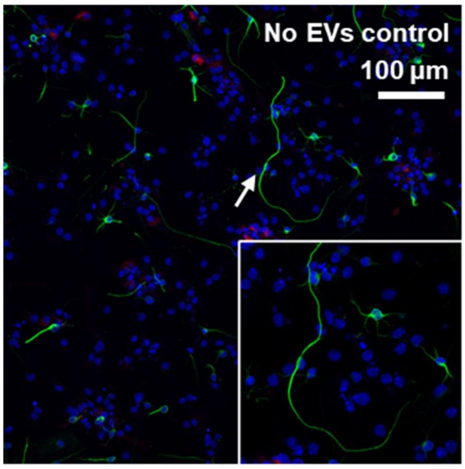


**Supplementary Figure S2:** Immunofluorescence images of no EV control showing differentiating neural cells in vitro. βIII-Tubulin (green), GFAP (red) and DiD (yellow) with DAPI counterstain (blue).
